# Supplementary material for: Histomorphometric Analysis of 38 Giant Cell Tumors of Bone after Recurrence as Compared to Changes Following Denosumab Treatment
Source: Cancers (Basel). 2023 Aug 24;15(17):4249. doi: 10.3390/cancers15174249 (PMC10486357; doi:10.3390/cancers15174249)
Supplement: Supplementary file 1 [file cancers-15-04249-s001.zip › Supplement Figure S1.pdf]

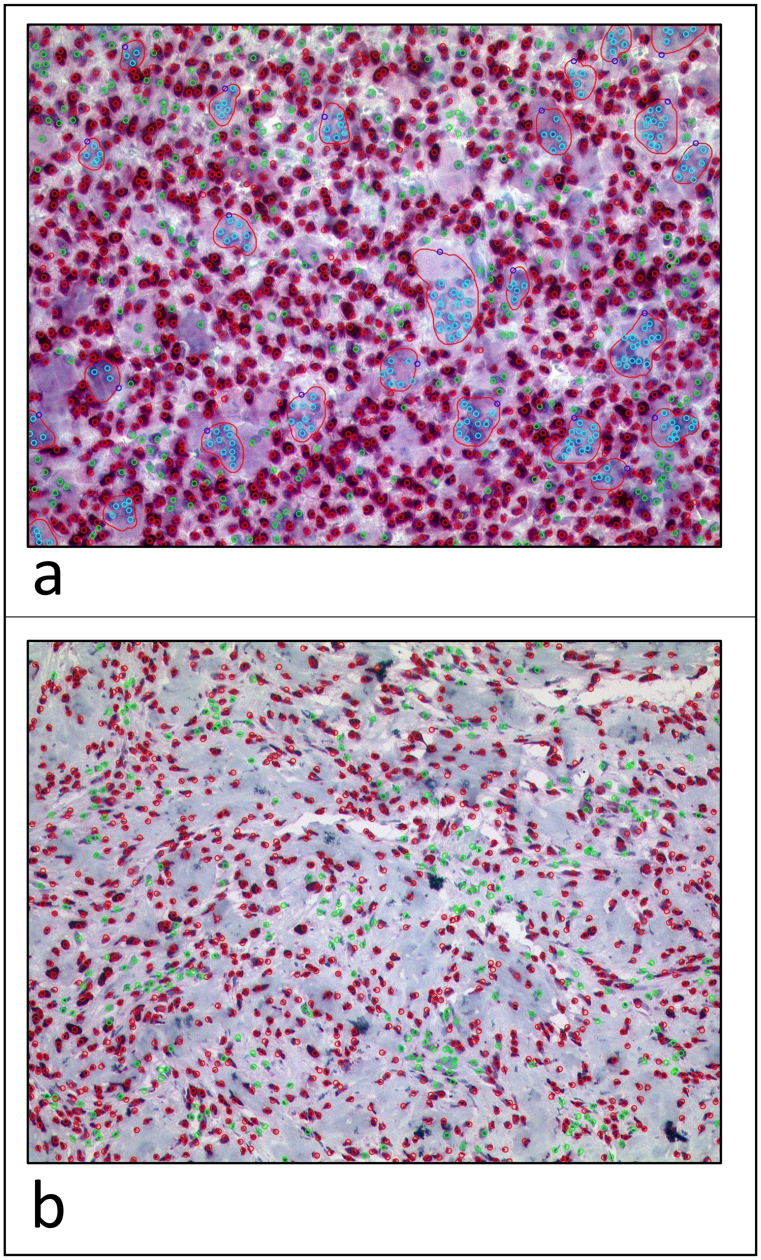

Supplement Figure S1: Demonstrating cell counting with open-source software QuPath. Colour coding: blue: giant cells; cyan: giant cell nuclei; red: H3F3A G34W stained cells; lime: unstained cells. Two examples: (a) before denosumab (b) after denosumab
