# Supplementary material for: Histomorphometric Analysis of 38 Giant Cell Tumors of Bone after Recurrence as Compared to Changes Following Denosumab Treatment
Source: Cancers (Basel). 2023 Aug 24;15(17):4249. doi: 10.3390/cancers15174249 (PMC10486357; doi:10.3390/cancers15174249)
Supplement: Supplementary file 1 [file cancers-15-04249-s001.zip › Supplement Table S3.pdf]

sample	(a) G34W positive (and SD)	(a) G34W negative (and SD)	(a) Giant cells (and SD)	(b) G34W positive (and SD)	(b) G34W negative (and SD)	(b) Giant cells (and SD)
24	536 (60,899)	234 (13,736)	12,667 (5,793)	0 (0)	1067,667 (95,695)	0 (0)
25	456 (129,308)	622,333 (123,616)	10 (6,164)	0 (0)	971 (41,012)	0 (0)

Supplement Table S3: Mean of G34W stained cells, G34W negative stained cells and giant cells counted in three representative microscopic fields. Standard deviation (SD) in brackets. (a) Columns 2-4 showing the data in the samples before denosumab therapy. (b) Columns 5-7 in the samples of the sarcoma after malignant transformation after denosumab therapy.
