# Supplementary material for: Histomorphometric Analysis of 38 Giant Cell Tumors of Bone after Recurrence as Compared to Changes Following Denosumab Treatment
Source: Cancers (Basel). 2023 Aug 24;15(17):4249. doi: 10.3390/cancers15174249 (PMC10486357; doi:10.3390/cancers15174249)
Supplement: Supplementary file 1 [file cancers-15-04249-s001.zip › Supplement Table S4.pdf]

Sample	(a) KI-67 positive stained cells in %	(a) SATB2 positive stained cells in %	(a) RUNX2 positive stained cells in %	(b) KI-67 positive stained cells in %	(b) SATB2 positive stained cells in %	(b) RUNX2 positive stained cells in %
26	n.a.	n.a.	n.a.	n.a.	n.a.	n.a.
27	5	50	70	20	10	95
28	5	80	90	5	40	95
29	5	80	95	10	30	20
30	10	30	30	5	10	10
31	5	70	n.a.	5	30	90
32	5	n.a.	n.a.	5	n.a.	n.a.
33	5	10	90	5	20	80
34	5	30	10	5	60	80
35	1	40	60	1	n.a.	n.a.
36	5	10	90	5	60	95
37	5	5	60	5	10	50
38	2	10	90	5	5	70

Supplement Table S4: Percentage of cells immunhistochemically stained for KI-67, SATB2 and RUNX2. (a) Columns 2-4 showing the data in the samples of primary GCTBs (b) Columns 5-7 of recurrent GCTBs.
