# Supplementary material for: Histomorphometric Analysis of 38 Giant Cell Tumors of Bone after Recurrence as Compared to Changes Following Denosumab Treatment
Source: Cancers (Basel). 2023 Aug 24;15(17):4249. doi: 10.3390/cancers15174249 (PMC10486357; doi:10.3390/cancers15174249)
Supplement: Supplementary file 1 [file cancers-15-04249-s001.zip › Supplement Table S5.pdf]

sample	(a) KI-67 positive stained cells in %	(a) SATB2 positive stained cells in %	(a) RUNX2 positive stained cells in %	(b) KI-67 positive stained cells in %	(b) SATB2 positive stained cells in %	(b) RUNX2 positive stained cells in %
1	0,1	60	90	0,1	30	20
2	5	30	90	0	1	5
3	5	60	90	1	30	70
4	5	80	95	1	10	n.a.
5	3	70	90	1	20	60
6	1	50	n.a.	1	n.a.	n.a.
7	2	80	95	1	50	70
8	2	50	90	1	10	80
9	5	60	50	3	10	30
10	15	50	90	5	20	40
11	10	40	90	1	5	70
12	10	30	90	1	5	20
13	20	70	95	1	50	50
14	5	80	95	0,1	5	50
15	5	60	95	1	20	80
16	10	70	90	5	15	n.a.
17	10	60	95	1	10	50
18	1	60	80	1	50	40
19	5	40	90	1	10	5
20	10	60	95	1	10	80
21	5	70	95	1	20	30
22	5	n.a.	n.a.	1	n.a.	n.a.
23	5	10	30	1	20	100

Supplement Table S5: Percentage of cells immunhistochemically stained for KI-67, SATB2 and RUNX2. (a) Columns 2-4 showing the data in the samples of GCTB before denosumab therapy (b) Columns 5-7 after denosumab therapy.
