# Supplementary material for: Histomorphometric Analysis of 38 Giant Cell Tumors of Bone after Recurrence as Compared to Changes Following Denosumab Treatment
Source: Cancers (Basel). 2023 Aug 24;15(17):4249. doi: 10.3390/cancers15174249 (PMC10486357; doi:10.3390/cancers15174249)
Supplement: Supplementary file 1 [file cancers-15-04249-s001.zip › Supplement Table S6.pdf]

Sample	(a) KI-67 positive stained cells in %	(a) SATB2 positive stained cells in %	(a) RUNX2 positive stained cells in %	(b) KI-67 positive stained cells in %	(b) SATB2 positive stained cells in %	(b) RUNX2 positive stained cells in %
24	5	n.a.	n.a.	60	0	10
25	5	50	60	50	80	90

Supplement Table S6: Percentage of cells immunhistochemically stained for KI-67, SATB2 and RUNX2. (a) Columns 2-4 showing the data in the samples of GCTB before denosumab therapy (b) Columns 5-7 of GCTBs after malignant transformation after denosumab therapy.
